# Ultra-hypofractionation versus standard hypofractionation in post-mastectomy radiotherapy (PMRT): a prospective randomised dosimetric and clinical comparison study

**DOI:** 10.3332/ecancer.2026.2151

**Published:** 2026-06-18

**Authors:** Meghal Prajapati, Anil Kumar Goel, Yamini Patel, Divyeshkumar Rana, S Lokesh, Pooja Panchal, Dhruv Rathod, Chandramouli Ramalingam, Kondeti Ajay Kumar

**Affiliations:** 1 Department of Radiation Oncology, Medical College Baroda, S.S.G. Hospital in Vadodara, Vadodara, India; 2 Department of Radiation Oncology, All India Institute of Medical Sciences (AIIMS), Bathinda, India; 3 Department of Radiation Oncology, All India Institute of Medical Sciences (AIIMS), Bibinagar, India

**Keywords:** breast cancer, post mastectomy radiotherapy, hypofractionation, ultra-hypofractionation

## Abstract

**Background:**

Post-mastectomy radiotherapy (PMRT) significantly reduces loco-regional recurrence and improves survival in breast cancer patients with node-positive or high-risk disease. The UK START trials established 40 Gy in 15 fractions as a safe and effective alternative to 50 Gy/25. The FAST-Forward trial later demonstrated non-inferiority of 26 Gy in 5 fractions, but only a minority of participants had undergone mastectomy. Limited prospective data exist for ultra-hypofractionated PMRT, particularly in the Indian setting, where shorter regimens could reduce patient and system burden.

**Methods:**

This prospective randomised study enrolled 50 women with stage II–IIIA invasive breast cancer treated with modified radical mastectomy. Patients were randomised equally to receive PMRT as either 40 Gy in 15 fractions (Group A) or 26 Gy in 5 fractions (Group B). CT-based planning was performed using 3D conformal radiotherapy or volumetric-modulated arc therapy techniques, with the deep inspiration breath hold (DIBH) technique implemented for left-sided cases to minimise cardiac dose.

**Results:**

Baseline demographics were balanced, though Group B had more left-sided tumours (68% versus 40%) and fewer node-positive cases (44% versus 72%). Both regimens achieved excellent planning target volume coverage (>95%). Ultra-hypofractionation (26 Gy/5) yielded lower ipsilateral lung exposure (V30 = 17% versus Group A V20 = 27%), reduced contralateral lung (300 versus 384 cGy), contralateral breast (247 versus 320 cGy) and spinal cord dose (938 versus 2,206 cGy). Mean heart dose was slightly higher in Group B (275 versus 181 cGy), but no patient exceeded a mean heart dose of 6 Gy, and all had V 25% <5%, consistent with international tolerance thresholds. Acute toxicity was minimal in both arms, limited to Grade 1 dermatitis; no Grade ≥2 toxicity was observed. At 6 months, all patients were alive and disease-free.

**Conclusion:**

Ultra-hypofractionated PMRT (26 Gy/5) is feasible, well-tolerated and dosimetrically advantageous for lungs, contralateral breast and spinal cord. Incorporating DIBH ensures safe heart sparing in left-sided disease. A longer follow-up is warranted to validate late toxicity and survival outcomes.

## Introduction

Breast cancer is the most prevalent type of cancer among women globally, with around 2.3 million new diagnoses reported in 2020 [1]. In India, it has overtaken cervical cancer as the leading cancer among women, showing a consistent increase in incidence in both urban and semi-urban areas [2, 3]. Patients in India tend to be diagnosed at a younger age and at more advanced stages compared to their counterparts in Western countries [4]. Since breast-conserving surgery is less common, modified radical mastectomy (MRM) remains the primary surgical method, making post-mastectomy radiotherapy (PMRT) an essential part of treatment in the Indian scenario [5].

### PMRT and the move toward hypofractionation

PMRT plays a crucial role in lowering locoregional recurrence rates and enhancing breast cancer–specific survival for patients with node-positive or high-risk disease [6]. The meta-analysis by the Early Breast Cancer Trialists’ Collaborative Group established that PMRT reduces 10-year recurrence rates by nearly 50% and boosts survival rates [7]. Historically, adjuvant radiotherapy has been administered as 50 Gy in 25 fractions over a span of 5 weeks [8]. Although this approach is effective, it demands considerable resources and poses inconvenience for patients, particularly in low- and middle-income countries like India, where access to radiotherapy is still limited [9, 10].

The UK START A and B trials demonstrated that delivering 40 Gy in 15 fractions over 3 weeks is equivalent to 50 Gy in 25 fractions in terms of tumour control and late toxicity [11]. With more than a decade of follow-up data, hypofractionation was found to be not only safe but possibly superior concerning late normal tissue effects. Subgroup analyses that included PMRT patients affirmed its relevance beyond breast-conserving therapy [12]. As a result, the regimen of 40 Gy/15 has become the international standard for both breast-conserving and PMRT in numerous Western institutions [13].

### The FAST-Forward trial and ultra-hypofractionation

The FAST-Forward trial (2020) further questioned the traditional treatment schedule by investigating ultra-hypofractionated regimens. More than 4,000 patients were randomised to receive either 40 Gy/15 or 26 Gy/5, administered over 1 week [14]. At the 5-year mark, the rate of ipsilateral breast tumour relapse was 2.1% for the 26 Gy/5 regimen compared to 1.7% for the 40 Gy/15, satisfying the criteria for non-inferiority [15]. Late toxicity and patient-reported outcomes were found to be similar [16]. Nonetheless, only about 10% of the participants in the FAST-Forward trial underwent mastectomy and detailed analyses focusing on PMRT were not reported separately [17]. Thus, the relevance of the FAST-Forward regimen for PMRT patients remains unclear.

PMRT differs from breast-conserving therapy in terms of target volume and complexity [18]. The treatment fields typically include the chest wall, mastectomy scar and often regional nodes (such as supraclavicular ± axillary). A tissue-equivalent bolus is usually applied to guarantee sufficient surface dosage, which can elevate acute skin toxicity [19]. Organs at risk (OARs) – including the heart, lungs, contralateral breast and spinal cord – are exposed to higher radiation levels because of the larger treatment fields. For patients with left-sided disease, preserving cardiac health is crucial, as studies showed a 7.4% increase in significant coronary events per Gy of mean heart dose [20]. Therefore, although ultra-hypofractionation presents significant convenience, its safety for PMRT requires confirmation through dosimetric and clinical studies.

### Indian context and evidence gap

India encounters considerable hurdles in providing radiotherapy services. The availability of machines is low, and waiting times for treatment can span several weeks. Patients often travel great distances, facing high out-of-pocket expenses and logistical challenges [21]. Shorter treatment regimens like 26 Gy/5 could yield significant benefits – shortening treatment duration, enhancing compliance and optimising the use of limited resources.

Nonetheless, evidence from Indian centers is still scarce. Several institutions have confirmed the safety and efficacy of 40 Gy/15 in PMRT [22, 23]. Small series conducted during the pandemic have investigated 26 Gy/5 in mixed cohorts; however, these studies were retrospective and included both breast-conserving and mastectomy cases [24]. A crucial evidence gap persists regarding the application of ultra-hypofractionated PMRT in Indian patients, particularly in terms of dosimetry, acute toxicity and early disease control.

This study was designed to fill the existing gap and was conducted as a prospective randomised trial. We assessed two widely used hypofractionated PMRT regimens – 40 Gy delivered in 15 fractions and 26 Gy in 5 fractions – in patients who had mastectomies for stage II–IIIA breast cancer. The main aim was to analyse the dosimetric differences in target coverage and doses to OAR. Secondary endpoints included evaluating acute toxicity and loco-regional control at the 6-month mark. By offering prospective data specific to India, this study seeks to determine whether the ultra-hypofractionated FAST-Forward regimen can be implemented safely and effectively in the PMRT context.

### Material and methods

#### Study design and setting

This clinical study was designed as a prospective, randomised, open-label trial carried out at the Department of Radiation Oncology, Medical College Baroda and S.S.G. Hospital in Vadodara, India, from January 2022 to March 2025. The Institutional Ethics Committee for Biomedical and Health Research (IECBHR) granted approval for the study (No. IECBHR/152-2022), and all participants provided written informed consent before joining. The primary aim of the trial was to evaluate and compare the dosimetric characteristics, acute toxicity and early clinical results of ultra-hypofractionated PMRT (26 Gy in 5 fractions over 1 week) against standard hypofractionated radiotherapy (40 Gy in 15 fractions over 3 weeks).

#### Eligibility criteria

The inclusion criteria encompassed:

- Histologically confirmed invasive breast carcinoma, stage II–IIIA (according to AJCC 8th edition).
- Completion of an MRM along with axillary clearance.
- ECOG performance status of 0–2.
- Age range of 18–70 years.
- Sufficient organ function to undergo adjuvant radiotherapy.

The exclusion criteria included:

- Prior radiotherapy delivered to the chest or thorax.
- Presence of metastatic disease identified during baseline staging.
- Incomplete surgical resection (indicated by positive margins).
- Previous neoadjuvant chemotherapy or systemic therapies modifying standard timelines.
- Presence of connective tissue disorders (such as systemic lupus erythematosus, scleroderma).
- Pregnancy or breastfeeding.

#### Randomisation and group allocation

A total of 50 eligible participants were randomly assigned to two treatment groups in a 1:1 ratio through block randomisation, employing a block size of 4 and was stratified by side (right versus left) [25]. A statistician who was not involved in the treatment delivery generated randomisation codes. The allocation remained concealed until the treatment planning stage.

**Group A (Standard hypofractionation):** 40 Gy administered in 15 fractions over a period of 3 weeks (2.67 Gy for each fraction).

**Group B (Ultra-hypofractionation):** 26 Gy given in 5 fractions across 1 week (5.2 Gy per fraction).

Both treatment groups were administered using linear accelerators equipped with 6 MV photons employing either 3D conformal radiotherapy (3DCRT) or volumetric-modulated arc therapy (VMAT), based on the anatomy of the patient and the preference of the treating physician.

#### Simulation and immobilisation

Patients underwent CT simulation in a supine position using a breast board, positioning both arms abducted above their heads and supported by a headrest. Attention was given to reproducible positioning, with tattoos marking reference points. Axial CT images were taken with a 3 mm slice thickness, capturing the area from the mandible to the upper abdomen and including the chest wall, lungs and heart. In certain cases, intravenous contrast was administered to help delineate vascular structures.

For cases involving the left side, the deep inspiration breath hold (DIBH) method was implemented to reduce exposure to the heart [26]. Patients were trained to achieve reproducible breath-holds during both simulation and treatment phases, using respiratory monitoring or surface-guided imaging to confirm consistent target positioning and optimal cardiac protection.

#### Contouring and target volumes

Target delineation was conducted based on the contouring guidelines established by ESTRO [27] and RTOG [28]:

- Clinical target volume (CTV): This included the chest wall, mastectomy scar and relevant regional nodes (including the supraclavicular fossa, infraclavicular and axillary levels I–III if necessary). Internal mammary nodes were included only when found to be pathologically involved.
- Planning target volume (PTV): This was generated by expanding the CTV by 5 mm in all directions, also cropped by 3 mm from the skin’s surface except in areas where bolus application was intended.
- Bolus application: A 0.5 cm tissue-equivalent bolus was utilised on alternate days for chest wall treatments to guarantee an adequate surface dose; the use of bolus was tailored to individual cases, particularly for those with skin involvement or high-risk features.

**OARs:** The ipsilateral and contralateral lungs, heart, contralateral breast, esophagus, brachial plexus and spinal cord were outlined in accordance with established consensus guidelines.

#### Treatment planning

The planning for treatment was conducted using Eclipse™ TPS (Varian) or Monaco™ TPS (Elekta).

For 3DCRT, tangential photon fields were utilised for the chest wall, employing the field-in-field technique to enhance homogeneity. Supraclavicular fields were aligned with appropriate shielding. In the case of VMAT, dual arcs with avoidance sectors were implemented, enhancing conformity while reducing dose to OARs.

Plans were adjusted so that a minimum of 95% of the PTV received the prescribed dose. Hotspots exceeding 107% were avoided. Dose-volume histograms were created for all targets and OARs.

#### Dosimetric evaluation

The following dosimetric endpoints were assessed and documented for each treatment plan: PTV: V95 (the volume receiving at least 95% of the prescribed dose), D_mean_ and D_max_.

Ipsilateral lung: V20 (for the 40 Gy/15 arm) and V30 (for the 26 Gy/5 arm), along with mean dose.

Heart: Mean dose, V25 (% of volume receiving 25 Gy or more).

Contralateral lung: Mean dose.

Contralateral breast: Mean dose.

Spinal cord: Dmax.

#### Treatment delivery

All patients were treated using 6 MV linear accelerators, with daily imaging guidance. Verification was conducted utilising kV imaging or cone-beam CT before each treatment fraction. Patients were evaluated weekly by radiation oncologists and nursing staff for toxicity monitoring. Adherence to the bolus application and treatment setup was recorded.

#### Toxicity assessment and follow-up

Toxicity was evaluated clinically on a weekly basis during radiation therapy and again at 1, 3 and 6 months following treatment completion. Acute toxicities were graded according to the Common Terminology Criteria for Adverse Events (v5.0), focusing on skin, lung, esophagus and hematologic factors.

Skin toxicity: erythema, desquamation, pigmentation changes and edema.

Pulmonary toxicity: symptoms such as cough and dyspnea, along with radiological changes noted on chest X-ray or CT scans.

Cardiac toxicity: Baseline ECG and echocardiograms were performed; any new symptoms were assessed clinically.

Follow-up included a physical examination, palpation of the chest wall and nodal beds and a thorough systemic review. Imaging (CECT chest or PET-CT) was conducted at baseline and at the 6-month mark to verify disease status.

#### Clinical outcomes

The following outcomes were documented:

Loco-regional control: absence of recurrence in the chest wall or supraclavicular nodes.

Distant metastasis-free survival: absence of systemic recurrence.

Overall survival: patients still alive at the time of the last follow-up.

Due to the limited follow-up duration, emphasis was placed on acute toxicity and early disease control rather than late-stage outcomes.

#### Statistical analysis

Data analysis was carried out using SPSS v26. Continuous variables were presented as mean ± SD or median (range) and comparisons were made using Student’s *t*-test or Mann–Whitney *U* test. Categorical variables were compared using chi-square or Fisher’s exact test, with *p*-values of <0.05 considered statistically significant. A subgroup analysis was carried out based on laterality (left versus right) to assess its effect on heart dosages. Dosimetric outcomes were compared across groups using normalised dose parameters to accommodate differences in fraction sizes.

#### Study workflow

**Figure 1.**
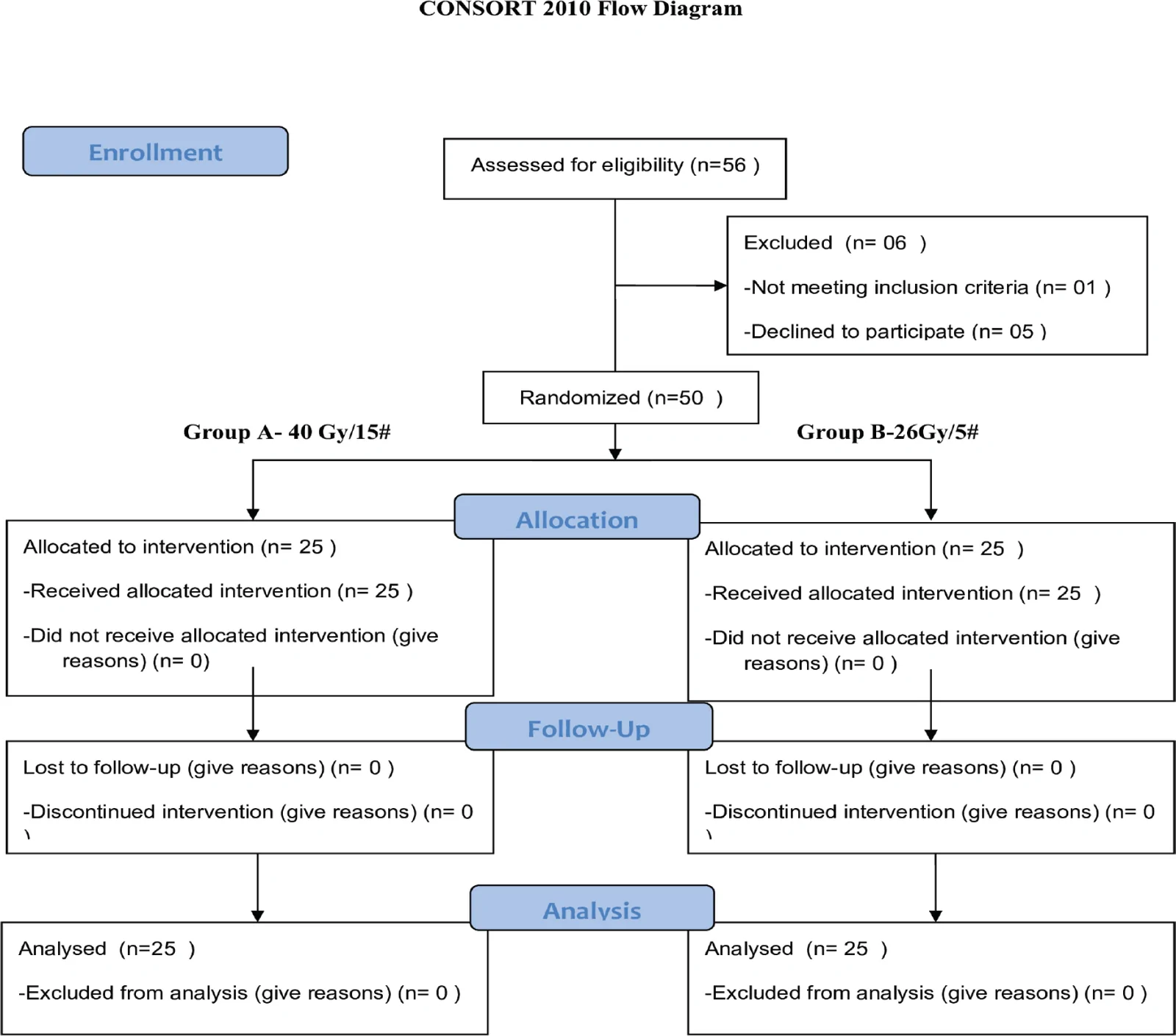
CONSORT 2010 flow diagram.

The entire workflow is depicted in a CONSORT-style diagram [29] (Figure 1):

Screening & eligibility: 56 patients evaluated → 50 deemed eligible.

Randomisation: 25 assigned to 40 Gy/15 (Group A), 25 to 26 Gy/5 (Group B).

Treatment planning: CT simulation, contouring, planning and quality assurance.

Treatment delivery: 3DCRT or VMAT with daily image-guided radiotherapy and DIBH.

Follow-up: Weekly reviews during treatment and post-treatment evaluations at 1, 3 and 6 months.

Endpoints: Dosimetry, assessment of acute toxicity and local and systemic control at the 6-month mark.

## Results

### Patient demographics and baseline characteristics

A total of 50 patients with stage II–IIIA breast cancer were equally randomised into two treatment groups: 25 underwent standard hypofractionated PMRT (40 Gy/15 fractions, Group A) and 25 received ultra-hypofractionated PMRT (26 Gy/5 fractions, Group B). In Group A, the median age was 48 years (range 38–58), while in Group B it was 50 years (range 35–66). Approximately one-third of the patients in both groups were under 40 years, emphasising the younger demographic associated with breast cancer in this cohort. All patients were female and underwent MRM with axillary lymph node dissection (Table 1).

Tumour grade distribution showed a higher prevalence of high-grade tumours: 60% grade 3 in Group A compared to 72% in Group B. When assessed by risk factors, 52% in Group A and 68% in Group B were identified as high-risk (age >50, grade 3). The laterality of tumours varied between the groups: right-sided tumours were more common in Group A (60%), while left-sided tumours were predominant in Group B (68%). This difference in laterality was taken into account when analysing cardiac dose metrics.

Nodal positivity occurred in 72% of patients in Group A, compared to 44% in Group B, indicating a slightly advanced nodal disease in the standard hypofractionation group. The majority of histological findings were invasive ductal carcinoma (80% in Group A, 72% in Group B), with the remainder being lobular carcinoma. The tumour stage distribution indicated that pT2 tumours were the most frequent (56% in Group A, 72% in Group B), followed by pT3 tumours.

All patients had negative margins, without residual disease. Lymphovascular invasion (LVI) was noted in 64% of Group A and 48% of Group B. All patients underwent adjuvant chemotherapy, with 52% starting on endocrine therapy. No patients received neoadjuvant chemotherapy.

### Dosimetric outcomes

#### Target volume coverage

Both treatment regimens provide excellent PTV coverage. The mean V95 was 97.4% in Group A and 95.6% in Group B. The homogeneity indices were slightly better in Group A, but coverage in both groups surpassed protocol requirements (>95%) (Tables 2 and 3).

#### Ipsilateral lung

In Group A, the mean ipsilateral lung V20 was 27%, whereas Group B had a mean V30 of 17%. While these parameters differ, both indicate high-dose lung volumes and show that Group B had significantly lower lung exposure. Mean lung doses were also reduced in the ultra-hypofractionated group (approximately 260 cGy compared to 320 cGy). These findings suggest enhanced lung sparing with the 26 Gy/5 regimen.

#### Contralateral lung

The mean dose to the contralateral lung was 384 cGy for Group A and 300 cGy for Group B. The decreased scatter dose observed with ultra-hypofractionation may lead to a reduced risk of radiation pneumonitis and long-term lung complications.

#### Heart

The mean heart dose stood at 181 cGy in Group A, increasing to 275 cGy in Group B. This somewhat elevated dose in Group B can be attributed to the higher prevalence of left-sided tumours (68%) in that group. Notably, no patient surpassed a mean heart dose of 6 Gy, and all maintained V 25% <5%, adhering to international safety standards. Employing heart-sparing techniques is particularly crucial when utilising 26 Gy/5 in left-sided PMRT.

#### Contralateral breast

The average dose to the contralateral breast was 320 cGy in Group A compared to 247 cGy in Group B. The reduced dose to the contralateral breast with the 26 Gy/5 regimen is clinically significant in decreasing the long-term risk of secondary cancers, especially in this relatively young patient demographic.

#### Spinal cord

The maximum dose to the spinal cord (D_max_) was significantly less in the ultra-hypofractionated group: 938 cGy in Group B versus 2206 cGy in Group A. This dosimetric benefit is due to smaller field sizes and diminished scatter associated with fewer treatment fractions.

#### Acute toxicity

The most frequently observed acute toxicity was skin reactions. In Group A, 68% of patients exhibited Grade 1 dermatitis, while around 12% experienced Grade 2 erythema. Group B displayed a similar trend, with approximately 70% showing Grade 1 dermatitis and rare occurrences of Grade 2 responses. No Grade ≥3 toxicity was noted in either group. Pigmentation alterations persisted in 15%–20% of patients after 3 months but were mild.

Other toxic effects, such as edema, esophagitis, brachial plexopathy and pneumonitis, were not reported in either group during the 12-month monitoring period.

#### Disease-free survival at 12 months

After a median follow-up of 12 months, all patients remained alive and free of disease. No recurrences in the chest wall were noted in either group. There were no recorded failures in the supraclavicular or axillary lymph nodes. No distant metastases were identified. Imaging with CECT or PET-CT verified the absence of relapse in all participants.

Overall, the findings indicate that ultra-hypofractionated PMRT (26 Gy/5) is both feasible and safe, offering beneficial dosimetric advantages for the lungs, contralateral breast and spinal cord, while showing early clinical outcomes comparable to standard hypofractionation.

## Discussion

This prospective randomised trial evaluated ultra-hypofractionated PMRT employing the FAST-Forward schedule (26 Gy in 5 fractions) against the current standard hypofractionation (40 Gy in 15 fractions) in 50 women diagnosed with stage II–IIIA breast cancer following MRM. Our results reveal that both treatment regimens achieved excellent coverage of the target volume, minimal acute side effects and a 100% disease-free survival rate at 6 months. Notably, ultra-hypofractionation offered significant dosimetric benefits in sparing the lungs, contralateral breast and spinal cord, with only a slight increase in the mean heart dose, largely due to a discrepancy in laterality between the groups.

### Comparison with FAST-Forward trial

The FAST-Forward trial was a pivotal phase III investigation that randomised more than 4,000 patients into three treatment groups: 40 Gy/15 or 27 Gy/5 or 26 Gy/5 [14]. At the 5-year mark, the rates of ipsilateral breast tumour relapse were 2.1% for the 26 Gy/5 group compared to 1.7% for the 40 Gy/15 group, confirming non-inferiority [15]. The toxicity profiles were similar, and the patient-reported outcomes were comparable between the two arms [16]. However, only about 10% of the participants in the FAST-Forward trial had received a mastectomy and detailed subgroup analyses specific to PMRT were not provided separately [14]. Our investigation directly addresses this oversight, zeroing in on patients who underwent mastectomy and showcasing feasibility, safety and advantageous dosimetry with 26 Gy/5 in the PMRT context.

An area of particular interest is the impact of bolus use, which is often necessary in PMRT to ensure sufficient skin dosage. There are concerns that administering five large fractions could worsen skin toxicity when a bolus is utilised [30]. Nonetheless, in our cohort, the occurrence of acute dermatitis was minimal and predominantly confined to Grade 1, with only two patients experiencing Grade 2 erythema in both groups. These results are consistent with findings from smaller sub-analyses of the FAST-Forward trial, which suggest that toxicity in chest wall cases is not significantly elevated [16].

### Comparison with the START trials

The START A and B trials laid the groundwork for hypofractionated breast radiotherapy. Following over 10 years of follow-up, both trials confirmed equivalence between 50 Gy/25 and 40 Gy/15, noting favourable late normal tissue outcomes in the hypofractionated groups. Critically, subsets of PMRT patients were included, demonstrating similar safety profiles [11]. Our research extends this evidence by examining an even shorter regimen, in line with the worldwide movement towards ultra-hypofractionation. The excellent coverage of the target area and minimal acute toxicity seen with 26 Gy/5 in our cohort provide confidence that the advantages of hypofractionation apply to PMRT populations as well.

### Dosimetric insights

Dosimetric evaluation highlighted several significant benefits of the 26 Gy/5 regimen. Exposure to the ipsilateral lung was decreased, with Group B displaying a V30 of 17% compared to Group A’s V20 of 27%. While direct comparisons of V20 and V30 across different fractionations can be complicated, these figures represent clinically significant reductions in high-dose exposure to lung tissue. Additionally, doses to the contralateral lung and breast were lower in the ultra-hypofractionated group, which may lead to a reduced risk of radiation pneumonitis and secondary cancers in the long term [31].

The maximal spinal cord dose was significantly lower with the 26 Gy/5 regimen (938 versus 2,206 cGy). Although neither approach reached tolerance limits, this observation underscores a consistent benefit of ultra-hypofractionation in minimising scatter and field spillover.

The sole parameter that was less favourable in Group B was the mean heart dose, which was elevated (275 versus 181 cGy). However, this variance was predominantly attributed to the higher proportion of left-sided tumours in Group B (68% versus 40%). Importantly, all patients maintained mean heart doses below 6 Gy and V 25% under 5%, well within established international guidelines [32]. The application of heart-sparing techniques like DIBH could further reduce cardiac exposure in left-sided cases, especially when utilising ultra-hypofractionation [33].

### Acute toxicity and early clinical outcomes

Our analysis indicated minimal acute toxicity, with Grade 1 dermatitis being the most prevalent adverse effect. This aligns with results from the START and FAST-Forward trials, where acute skin toxicity remained modest and mostly self-resolving [11, 14]. There were no reports of Grade ≥3 toxicities, brachial plexopathy, esophagitis or pneumonitis during the short follow-up period.

At the 6-month follow-up, all patients were disease-free, with no loco-regional recurrences or distant metastases. Although further follow-up is necessary, these early results reinforce the oncological safety of the 26 Gy/5 regimen within the PMRT framework.

### Indian evidence and relevance

In India, the use of hypofractionation has gained popularity, with multiple institutional studies reporting positive results with a regimen of 40 Gy/15 in PMRT [22, 23]. During the COVID-19 pandemic, some institutions explored a regimen of 26 Gy/5 in breast cancer patients; however, these studies were retrospective, included diverse patient populations and lacked sufficient power to evaluate PMRT separately [24]. Our prospective randomised data represent one of the first systematic assessments of 26 Gy/5 specifically in PMRT within an Indian demographic.

The significance of ultra-hypofractionation is amplified by the limited resources available in Indian oncology. The availability of radiotherapy machines is insufficient compared to the number of patients, leading to lengthy waiting lists, which continue to be a significant issue [21]. Many patients must travel considerable distances from rural locations and incur substantial out-of-pocket expenses [34]. Cutting down PMRT from 3 weeks to 1 week could significantly boost compliance, alleviate financial stress and enhance the usage of limited infrastructure.

### Clinical implications

The results of this study advocate for the cautious adoption of ultra-hypofractionated PMRT, especially in settings with limited resources where reducing the treatment burden presents considerable benefits for both patients and healthcare systems. The dosimetric benefits noted for the lungs, contralateral breast and spinal cord indicate a potential decrease in late complications. Nonetheless, careful monitoring of cardiac exposure is necessary, particularly in left-sided cases. Regular incorporation of DIBH or advanced techniques like VMAT with cardiac sparing may be critical when administering 26 Gy/5.

### Limitations

The primary limitations of this study include a small sample size, a single-center design and a relatively brief follow-up period of 6 months. Long-term outcomes, especially concerning late cardiac issues, lung fibrosis, brachial plexopathy and cosmetic results, cannot be evaluated at this time. Additionally, our trial was not designed to assess survival outcomes. Another constraint is the disparity in laterality between the study groups, which affected heart dose comparisons. Another key limitation is the comparison of dosimetric parameters between regimens with differing fractionation schedules. Due to underlying radiobiological differences, such comparisons may not accurately represent equivalent biological effects on OARs, as fractionation-dependent dose–response relationships are not yet fully established. Larger, multi-center studies with extended follow-up periods are necessary to validate these results.

### Future directions

Future investigations should concentrate on the long-term effects of 26 Gy/5 in PMRT, including late toxicities, survival rates and quality of life measures. Collaborative studies across multiple institutions in India would yield more widely applicable data and tackle the unique demographics and treatment challenges encountered in this population. The integration of advanced imaging, adaptive planning and heart-sparing strategies will be vital in optimising ultra-hypofractionation for PMRT.

## Conclusion

This prospective randomised study indicates that ultra-hypofractionated PMRT (26 Gy/5 fractions) is practical, safe and dosimetrically advantageous compared to 40 Gy/15, with minimal acute toxicity and favourable early outcomes. Although heart exposure was slightly elevated in left-sided cases, it remained within acceptable limits. This regimen provides significant logistical and resource-related advantages, making it particularly appealing in settings with limited resources. More extensive, long-term studies are needed to affirm the stability of tumour control and late toxicity outcomes.

## List of abbreviations

3D-CRT, 3-dimensional conformal radiotherapy; BCS, Breast conservation surgery; CTV, Clinical target volume; DIBH, Deep inspiratory breath hold; ER, Estrogen receptor; HER-2, Human epidermal growth factor receptor; IMRT, Intensity modulate radiotherapy; MLC, Multi leaf collimator; MRM, Modified radical mastectomy; MU, Monitor units; NCI-CTCAE, National cancer institute- common terminology criteria for adverse effect; OAR, Organs at risk; PMRT, Post-mastectomy radiotherapy; PR, Progesterone receptor; PTV, Planning target volume; VMAT, Volumetric modulated arc therapy; WBRT, Whole breast radiotherapy.

## Conflicts of interest

None.

## Funding

None.

## Ethical approval

The Institutional Ethics Committee for Biomedical and Health Research (IECBHR) of Medical College Baroda and S.

S.G. Hospital in Vadodara, India, granted approval for the study (No. IECBHR/152-2022) and all participants provided written informed consent before joining.

## Author contributions

AKG proposed the initial concept idea, conceived and designed the study protocol. MP and DKR contributed to the conceptualisation and conduction of the study. KAK and CR contributed to the drafting of the manuscript and critical revision of the manuscript for intellectual content. YP, LS, PP and DR contributed to the critical revision of the manuscript for important intellectual content. All authors discussed, advised and revised the manuscript. All authors read and accepted the final version.

## Data availability

The data and material of this study will be made available to the public upon request.

## Figures and Tables

**Table 1.** Demographic, clinical and treatment characteristics at randomisation (n = 50).

Characteristic	40 Gy in 15 fractions (n = 25)	26 Gy in 5 fractions (n = 25)
1. Age (years)		
Median (range)	48 (38–58)	50 (35–66)
<40	5 (20%)	5 (20%)
40–49	11 (44%)	10 (40%)
50–59	9 (36%)	7 (28%)
60–69	0	3 (12%)
≥70	0	0
2. Sex		
Female	25 (100%)	25 (100%)
Male	0	0
3.Tumour grade		
Grade 1	0	0
Grade 2	10 (40%)	7 (28%)
Grade 3	15 (60%)	18 (72%)
4. Risk group		
Low (age <50, grade 1/2)	12 (48%)	8 (32%)
High (age ≥50, grade 3)	13 (52%)	17 (68%)
5. Primary surgery		
MRM	25 (100%)	25 (100%)
Axillary dissection	25 (100%)	25 (100%)
6. Side of lesion		
Right	15 (60%)	8 (32%)
Left	10 (40%)	17 (68%)
7. Pathological node status		
Positive	18 (72%)	11 (44%)
Negative	7 (28%)	14 (56%)
8. Histological subtype		
Invasive ductal	20 (80%)	18 (72%)
Lobular	5 (20%)	7 (28%)
Mixed/others	0	0
9. Pathological tumour stage		
T1 (Tmi–T1c)	3 (12%)	2 (8%)
T2	14 (56%)	18 (72%)
T3	8 (32%)	5 (20%)
10. Resection margin (pR status)		
Negative	25 (100%)	25 (100%)
11. LVI ￼		
Positive	16 (64%)	12 (48%)
Negative	9 (36%)	13 (52%)
12. Neoadjuvant chemotherapy		
None	25 (100%)	25 (100%)
13. ER/HER2 status		
ER+/HER2+	8 (32%)	5 (20%)
ER−/HER2+	2 (8%)	1 (4%)
ER+/HER2−	5 (20%)	8 (32%)
ER−/HER2− (Triple negative)	10 (40%)	11 (44%)
14. Adjuvant chemotherapy		
Yes	25 (100%)	25 (100%)
15. Endocrine therapy		
Yes	13 (52%)	13 (52%)
No	12 (48%)	12 (48%)
16. Boost given (10 Gy/5 fractions)		
No	25 (100%)	25 (100%)

**Table 2.** Group A – 40 Gy in 15 fractions over 3 weeks.

Sr No.	Age	Diagnosis	Side	Stage	PTV		Ipsilateral lung		Contralateral lung	Heart		Contralateral breast	Spinal cord	Skin reaction				Disease free survival
					95%	105%	V2OGy	Dmean	Dmean	V25%	Dmean	Dmean	Dmax	Post RT	1 month	3 months	6 months	
1	44	Ca Breast (PO)	Right	pT_2_N_1a_M_0_	96.70%	4%	27.50%	1,226.2	294	4.90%	531.4	397.9	2,675.2	1	1	0	0	6 months
2	52	Ca Breast (PO)	Left	pT_2_N_1a_M_0_	97.70%	1.60%	26.70%	1,323	319.5	2.40%	636.8	320.2	2,645.4	0	0	0	0	6 months
3	39	Ca Breast (PO)	Left	pT_2_N_1a_M_0_	97.50%	1.60%	27.33%	1,342.5	256.1	8.60%	559.8	342.6	3,021.5	1	0	0	0	6 months
4	45	Ca Breast (PO)	Right	pT_2_N_1a_M_0_	97.10%	0.70%	16.34%	1,779.6	69.2	3.90%	367.4	75.5	3,336.6	0	0	0	0	6 months
5	54	Ca Breast (PO)	Left	pT_3_N_0_M_0_	98.90%	0.01%	24.42%	1,630	41.6	8.81%	581.8	136	542.2	0	0	0	0	6 months
6	43	Ca Breast (PO)	Left	pT_3_N_0_M_0_	99.20%	10.40%	30.38%	1,459.1	36.8	3.10%	582.6	81.6	106.8	1	0	0	0	6 months
7	38	Ca Breast (PO)	Right	pT_3_N_0_M_0_	99.20%	11.80%	29.88%	1,527.1	243.3	3.60%	477.1	340.7	2,049.8	1	0	0	0	6 months
8	56	Ca Breast (PO)	Right	pT_3_N_0_M_0_	99.80%	21.40%	26.50%	1,443.9	238.7	0.20%	317.5	217.8	1,912.6	0	0	0	0	6 months
9	58	Ca Breast (PO)	Right	pT_2_N_1a_M_0_	98.90%	11.70%	28.60%	1,328	254.4	2.10%	380	395.4	2,037.4	0	0	0	0	6 months
10	45	Ca Breast (PO)	Left	pT_2_N_1a_M_0_	97.40%	14.60%	32.00%	37.2	7.6	10.30%	12.1	9.8	47.3	3	1	0	0	6 months
11	49	Ca Breast (PO)	Right	pT_2_N_1a_M_0_	97.60%	19.10%	25.76%	1,330.1	329.4	0.20%	319.5	232.4	2,168	1	0	0	0	6 months
12	55	Ca Breast (PO)	Right	pT_2_N_1a_M_0_	90.10%	1.40%	23.86%	1,277.2	259.2	1.43%	350.4	272.4	2,193	0	0	0	0	6 months
13	48	Ca Breast (PO)	Left	pT_1_N_1a_M_0_	96.405	34.80%	27.72%	1,301.7	50.9	0.10%	30.7	46.9	3,996	1	0	0	0	6 months
14	38	Ca Breast (PO)	Right	pT_1_N_1a_M_0_	97.40%	1.10%	27.25%	1,256.7	34.3	0.10%	91.2	12.8	4,029.2	1	0	0	0	6 months
15	56	Ca Breast (PO)	Right	pT_3_N_0_M_0_	97.30%	0.255	30.62%	1,610.6	279.7	2.30%	523.3	295.4	2,335.6	3	1	0	0	6 months
16	42	Ca Breast (PO)	Right	pT_2_N_1a_M_0_	98.20%	2.60%	27.60%	1,472.5	156.2	3.30%	521.4	264.8	32,86	0	0	0	0	6 months
17	46	Ca Breast (PO)	Left	pT_2_N_1a_M_0_	97.70%	4.20%	28.50%	1,532.4	243.5	4.60%	434.6	184.8	3,328.9	0	1	0	0	6 months
18	53	Ca Breast (PO)	Left	pT_2_N_1a_M_0_	98.70%	3.40%	29.10%	1,284.5	323.4	7.50%	381.9	364.5	2,434.4	1	0	0	0	6 months
19	48	Ca Breast (PO)	Right	pT_3_N_0_M_0_	97.70%	1.50%	30.50%	1,473.5	189	4.60%	432.4	345.8	4,021.3	0	0	0	0	6 months
20	54	Ca Breast (PO)	Right	pT_2_N_1a_M_0_	96.60%	5.50%	30.30%	1,456	254.9	1.40%	327.9	323.5	4,098.1	0	0	0	0	6 months
21	39	Ca Breast (PO)	Left	pT_3_N_0_M_0_	98.10%	3.60%	27.90%	1,546.3	432.1	6.70%	263.8	128.7	2,674.8	1	1	0	0	6 months
22	57	Ca Breast (PO)	Right	pT_2_N_1a_M_0_	97.50%	1.20%	28.50%	1,634.2	323.5	3.40%	325.8	234.9	2,435.5	1	0	0	0	6 months
23	50	Ca Breast (PO)	Right	pT_3_N_0_M_0_	97.00%	3.10%	29.10%	1,432	278.9	2.10%	432.7	387.4	2,698.3	0	0	0	0	6 months
24	44	Ca Breast (PO)	Left	pT_2_N_1a_M_0_	98.30%	4.50%	23.10%	1,367.9	198.8	3.30%	212.4	234.5	2,298.2	3	0	0	0	6 months
25	41	Ca Breast (PO)	Right	pT_3_N_0_M_0_	97.40%	2.60%	24.40%	1,645.8	286.4	4.60%	434.2	127.4	2,987.3	0	0	0	0	6 months

**Table 3.** Group B – 26 Gy in 5 fractions over 1 week.

Sr No.	Age	Diagnosis	Side	Stage	PTV		Ipsilateral lung		Contralateral lung	Heart		Contralateral breast	Spinal cord	Skin reaction				Disease free survival
					95%	105%	V30%	Dmean	Dmean	V25%	Dmean	Dmean	Dmax	Post RT	1 month	3 months	6 months	
1	42	Ca Breast (PO)	Left	pT_2_N_0_M_0_	97.10%	17.80%	15.96%	272.2	247.8	1.35%	229.7	246.3	907	0	0	0	0	6 months
2	55	Ca Breast (PO)	Left	pT_2_N_1a_M_0_	95.60%	14.20%	16.57%	221.8	282.4	4.43%	382.5	269.2	584.3	0	0	0	0	6 months
3	45	Ca Breast (PO)	Left	pT_2_N_1a_M_0_	98.20%	19.40%	15.54%	300.9	371.1	4.63%	332	369.6	1,413.8	1	1	0	0	6 months
4	35	Ca Breast (PO)	Left	pT_2_N_0_M_0_	96.58%	50.30%	11.87%	312.2	262.8	5.02%	294.8	262.8	1,435.3	0	0	0	0	6 months
5	52	Ca Breast (PO)	Left	pT_2_N_0_M_0_	90.10%	17.96%	17.21%	615.3	240.7	4.70%	314.1	240.3	427.7	1	0	0	0	6 months
6	56	Ca Breast (PO)	Right	pT_3_N_0_M_0_	90.50%	23.55%	11.72%	705.3	384.3	3.59%	357.1	322.9	574.9	1	1	0	0	6 months
7	38	Ca Breast (PO)	Right	pT_1_N_1a_M_0_	97.10%	19.23%	16.79%	556	174.1	0.60%	163.3	225.2	373.8	1	0	0	0	6 months
8	66	Ca Breast (PO)	Right	pT_2_N_0_M_0_	90.28%	23.50%	14.60%	575.3	252.9	4.90%	320.9	322	454.1	1	0	0	0	6 months
9	46	Ca Breast (PO)	Left	pT_2_N_1a_M_0_	96.10%	18.90%	23.67%	566.4	264.6	4.10%	344.6	348.2	2,024.8	1	0	0	0	6 months
10	52	Ca Breast (PO)	Left	pT_1_N_1a_M_0_	97.10%	0.55%	12.64%	602.8	219	3.70%	225.5	192.5	1,214.5	1	0	0	0	6 months
11	65	Ca Breast (PO)	Left	pT_3_N_0_M_0_	98.20%	15.`10%	15.47%	521.8	271.1	3.02%	332	264.5	940.2	0	0	0	0	6 months
12	48	Ca Breast (PO)	Right	pT_2_N_0_M_0_	98.90%	14.60%	16.50%	612.6	234.4	4.22%	354.7	328.2	1,418.6	1	1	0	0	6 months
13	45	Ca Breast (PO)	Left	pT_2_N_1a_M_0_	95.60%	19.40%	21.24%	600.9	247.8	5.13%	294.8	246.7	587.8	0	0	0	0	6 months
14	62	Ca Breast (PO)	Left	pT_3_N_0_M_0_	97.60%	11.20%	16.10%	522.6	382.7	3.44%	235.4	320.6	450.8	0	0	0	0	6 months
15	48	Ca Breast (PO)	Left	pT_2_N_1a_M_0_	95.80%	22.20%	13.80%	606.8	282.4	4.50%	320.6	239.8	1,260.4	1	1	0	0	6 months
16	45	Ca Breast (PO)	Left	pT_2_N_1a_M_0_	97.10%	32.70%	22.50%	634.5	298.4	3.60%	356.9	199	1,098	0	0	0	0	6 months
17	47	Ca Breast (PO)	Right	pT_2_N_1a_M_0_	96.20%	25.60%	11.70%	324.6	250	2.30%	298.8	276.9	546	1	1	0	0	6 months
18	52	Ca Breast (PO)	Left	pT_2_N_1a_M_0_	95.90%	31.50%	11.20%	434.6	298.9	2.20%	337.9	378.9	756.9	0	0	0	0	6 months
19	54	Ca Breast (PO)	Right	pT_2_N_0_M_0_	98.30%	12.20%	16.30%	232.5	300	2.70%	287.9	321.9	698	0	0	0	0	6 months
20	48	Ca Breast (PO)	Left	pT_2_N_1a_M_0_	97.60%	16.80%	19.50%	234.6	253.5	3.90%	196.9	320.8	987.9	1	0	0	0	6 months
21	39	Ca Breast (PO)	Left	pT_2_N_1a_M_0_	97.10%	18.10%	18.70%	449.0	276.1	2.30%	384.9	278	653.9	0	1	0	0	6 months
22	41	Ca Breast (PO)	Right	pT_2_N_1a_M_0_	95.40%	27.70%	16.50%	471.9	387.7	3.90%	350.6	299.6	563.8	1	0	0	0	6 months
23	52	Ca Breast (PO)	Left	pT_2_N_0_M_0_	96.70%	19.20%	13.70%	357.3	211.9	1.50%	160.4	156.9	600	1	0	0	0	6 months
24	43	Ca Breast (PO)	Left	pT_2_N_1a_M_0_	97.60%	32.50%	15.10%	155.4	220.9	4.96%	244.6	298.6	765.9	0	0	0	0	6 months
25	49	Ca Breast (PO)	Left	pT_2_N_1a_M_0_	98.90%	16.30%	11.90%	352.5	184.7	3.70%	280	310	673	1	0	0	0	6 months
